# Correlation between air pollution and cognitive impairment among older individuals: empirical evidence from China

**DOI:** 10.1186/s12877-023-03932-z

**Published:** 2023-09-15

**Authors:** Huan Liu, Tiantian Hu

**Affiliations:** 1 School of Society, Soochow University, No. 199, Ren’ai Road, Suzhou Industrial Park, Su Zhou, Jiangsu 215123 China; 2 Shenzhen Futian District Economic Development Promotion Association, Shenzhen, China; 3 Qiaocheng Consulting (Shenzhen) Co., Ltd, Shenzhen, China

**Keywords:** Air pollution, Older individuals, Cognitive impairment, Depression, Mental state

## Abstract

**Background:**

Little information is available regarding the impact of air pollution on cognitive impairment in older individuals in developing countries. This study empirically tested the impacts of the air quality index (AQI), air pollution intensity (quantified by the number of days of extreme air pollution in a year), and different pollutants on the cognitive abilities of older Chinese individuals.

**Methods:**

A panel of 28,395 participants spanning 122 cities in 2015 and 2018 was used, based on 3-year follow-up survey data from the China Health and Retirement Longitudinal Study (CHARLS) database. Data from the two phases of the CHARLS microsurvey were combined with relevant statistical data on air pollution in each region in the current year. These two surveys were used to investigate changes in basic health and macro-environmental indicators in older individuals in China, and a mean difference test was conducted. We then reduced the sample selection error by controlling for environmental migration and used two-way fixed and instrumental variable methods for endogenous treatment to avoid the estimation error caused by missing variables.

**Results:**

Air pollution had a significantly negative effect on the cognitive abilities of older individuals (odds ratio [OR]: 1.4633; 95% confidence interval [95% CI]: 1.20899–1.77116). Different pollution intensities(only AQI value is greater than 200 or more) had apparent effects on cognitive impairment, with an OR of approximately 1.0. Sulfur dioxide had significantly negative effects on cognitive ability, with OR of 1.3802 (95% CI: 1.25779–1.51451). Furthermore, air pollution impact analysis showed heterogeneous results in terms of age, sex, education, and regional economic development level. In addition, social adaptability (calculated using social participation, learning, adaptability, and social support) not only had a significant positive effect on the cognitive abilities of older individuals, but also regulated the cognitive decline caused by air pollution.

**Conclusions:**

Air pollution affects cognitive impairment in older individuals, especially in those with lower education levels, and living in economically underdeveloped areas. This effect is synchronous and has a peak at an AQI of > 200.

## Background

Air pollution has become a global issue that causes long-term harm to physical and mental health. In terms of effects on physical health, studies have demonstrated that individuals who are exposed to air pollution for short or long periods may experience acute or chronic health problems [1]. Specifically, many studies have shown that nitrogen dioxide (NO_2_) [2], sulfur dioxide (SO_2)_ [3, 4], particulate matter (PM_10_ [5] and PM_2.5_ [6]), and other air pollutants, have significant negative impacts on people’s quality of life and mental health [7, 8]. Air pollution also increases the incidence of symptoms of depression [9]. Many studies on the health effects of air pollution are available, but few focus on the older population, with the majority focusing on the impact on the quality of life and overall health of older people [10, 11]. More studies are required to elucidate the impact of air pollution on the physical and mental health of older individuals, to improve the quality of life, and increase happiness in this demographic, eventually promoting and coordinating the development of an aging society and achieving the goal of healthy aging. As an essential component of the health of older individuals, cognitive impairment affects not only the quality of life but also the overall social vitality of a country or region. With the rapid aging of the population seen in many developed countries, cognitive impairment has become an increasingly serious health manifestation [10]. Therefore, recognizing the association between cognitive impairment in older individuals and air pollution is of great practical significance [4, 12, 13]. The association between cognitive impairment and air pollution has both practical and policy implications for older Chinese individuals.

According to the 2020 Global Environmental Performance Index (EPI) report jointly released by the Center for Environmental Law and Policy at Yale University and the Center for International Earth Science Information Network at Columbia University, China ranked 120th with a score of 37.3 among the 180 countries [14]. Air quality was the main factor that lowered the EPI in China. In a separate evaluation of air quality, China ranked 137th (with a score of 27.1), and its regional rank was 21st in the Asia–Pacific region, higher only than those of the Solomon Islands, Indonesia, Myanmar, and Mongolia. The rapid development of an aging population, as well as its large scale, underscore the prominence and the challenge surrounding China's elderly population. For example, by the end of 2020, China's population aged ≥ 65 years had reached 190 million, accounting for 13.5% of the total population [15].

Research on cognitive impairment in older individuals has been conducted in developed countries; however, relevant research in developing countries is insufficient. The existing research conclusions are relatively consistent — namely, that air pollution worsens cognitive impairment in older individuals, but the specific mechanism behind this has not been agreed upon. In addition, few studies have demonstrated the impacts of different pollution intensities on cognitive impairment in older individuals, and the impact of air pollution combined with the synergistic effects of natural and social environments has hardly been considered.

Based on the existing research, this study attempted to empirically test the impact of air pollution on the cognitive abilities of Chinese older individuals by using survey data from the China Health and Retirement Longitudinal Study (CHARLS) in the year 2015 and 2018. Regional macro-air pollution indicators and related pollutants were matched to relevant meteorological data. Compared to existing research, the main innovations of this study are as follows: (1) This study focused on older individuals (aged 60 and above) in China and investigated the impact of air pollution on cognitive levels in developing countries, thus filling gaps in the current research landscape, as most existing research has focused on cognitive impairment in older individuals only in developed countries; (2) this study not only investigated the impact of air pollution on cognitive impairment in older individuals, but also investigated the impact of different pollution intensities, as well as the internal mechanisms of air pollution affecting the cognitive abilities of older individuals; (3) we analyzed the impact of social adaptability as a proxy indicator of social environment, in order to test the impact of air pollution and social adaptability on the cognitive abilities of older individuals; and (4) in terms of specific index selection and variable processing, we used the air quality index (AQI) as a proxy index of air pollution, drawing on the experience of Sun and Gu (2008) and Wen and Gu (2012), to define seven air pollution levels using an air pollution index (API) [10, 11]. The number of days with cumulative extreme AQI values was selected as the duration of long-term exposure to a certain air pollution state, which was divided into five grades, and the impact of air pollution intensity on cognitive impairment in older individuals was investigated. This differs from the definitions used by Sun and Gu (2008) and Wen and Gu (2012) [10, 11].

### Theory and hypothesis

#### The impact of air pollution on the health of older individuals

The health effects of air pollution have been widely noted by researchers, but most have focused on the impact of air pollution on physical health [16, 17]. In recent years, researchers have investigated the relationship between air pollution and mental health. Individual cognitive impairment or mental health status was the core of this study. Studies have shown that long-term exposure to air pollution can significantly worsen the degree of depression in individuals and that this state persists over time [18]. Regarding the impact of different pollutants, studies have found that long-term exposure to fine particles enhances anxiety and that the short-term damage exerted by PM_2.5_ is greater than its long-term damage [19, 20]. Tian et al. found that SO_2_ emissions had a significant impact on mental health in older individuals, showing a positive "U-shaped" curve [21]. In addition, after adjusting for other confounding factors, short-term changes in pollutant levels and long-term exposure to traffic pollution are positively correlated with depression [22]. In terms of transmission mechanisms, studies have revealed that long-term exposure to air pollution increases the probability of chronic mental diseases and significantly increases the probability of mental disorders in people with physical health problems [20]. Research has also shown that air pollution affects the overall health of older individuals [10, 11]. In addition, significant research has been conducted to understand how air pollution relates to activities of daily living (ADL) and self-assessment of health-related and health-assessment indicators. For example, Zhang et al. found that air pollution can significantly affect ADLs in a wide segment of the population [23]. As the air quality deteriorates, people have been shown to employ measures such as traveling by bus or subway and decreasing walking and cycling. Stineman et al. found different states and characteristics of the ADLs of individuals at different stages, and these stages had strong associations with conditions that improved cognitive function in older individuals [24]. Corrêa et al. found that physical activity increases recognition function; in other words, physical activity can improve cognitive function in older individuals [25]. Jylhä built a model describing the health assessment process to show how self-rated health can reflect the state of the human body and mind [26]. Ju found that chronic exposure to air pollutants had significant negative effects on ADLs and self-rated health in middle-aged and older individuals [27]. Kato et al. found positive associations between positive attitudes toward life and self-rated health assessments and participants' current cognitive functions [28]. These results show that air pollution not only directly affects individuals’ ADL, self-rated health, and other proxy indicators but also indirectly affects individuals’ cognitive functions. Based on these facts, this study proposed the following hypothesis:

Hypothesis 1: Air pollution directly impacts the abilities of older individuals to perform ADL and self-rated health assessments and indirectly impacts their cognition.

Mechanism by which air pollution impacts cognitive impairment in older individuals.

Numerous studies have addressed cognitive impairment in older individuals from the perspective of the social sciences [20, 29, 30], but few discussions have been conducted from the perspective of the natural environment. For example, Jennifer et al. found that long-term exposure to PM_2.5–10_ and PM_2.5_, at levels typically experienced by many individuals in the United States, was associated with significantly worse cognitive decline in older women [31]. Exposure to long-term PM_2.5_ and NO_2_ was associated with decreased cognitive function in the older cohort, and individuals who experienced a stroke or elevated anxiety were more susceptible to the effects of PM_2.5_. Additionally, PM_2.5_ may impact cognition through pathways related to mood disorders [32]. Some researchers have also studied the cognitive abilities of older men (aged 60 and above) from the perspective of the traffic environment and whether ambient traffic-related air pollution is associated with decreased cognitive function [33]. By analyzing cognitive impairment in older women, Tamara et al. found that associated markers of air pollution were located in the visuospatial domain of the brain [34]. Exposure to air pollution increases the risk of inflammatory processes and cardiovascular mortality [4]. Therefore, such exposure may also be a risk factor for cognitive impairment or dementia. Peters et al. provided evidence of a potential association between air pollution and subsequent cognitive decline [35]. In addition, Zhang et al. found that long-term exposure to air pollution impedes cognitive performance on verbal and mathematics tests [36]. Damage to the aging brain due to air pollution is likely to lead to substantial health and economic costs, considering that cognitive function is critical for older individuals to run daily errands and make high-stake decisions. Jennifer et al. also stated that the association between PM_2.5_ and cognitive errors was more prevalent in older individuals living in high-stress neighborhoods [37]. These findings support recent theoretical developments in research on environmental health and health disparities, which emphasize the synergistic effects of neighborhood social stressors and environmental hazards on the health of residents [34, 35]. In addition, elevated PM pollution levels significantly affect short-term cognition. This implies that average human cognitive ability varies from city to city and country to country, as a function of PM air pollution exposure [38].

This study, therefore, proposed a second research hypothesis, which was also our core research topic.

Hypothesis 2: Air pollution has a significant direct impact on the cognitive abilities of older individuals, and as the pollution intensity increases, the impact of pollution also increases.

### Influence of social adaptability on cognitive impairment in older individuals

Social adaptability is a general term for the ability or capability to adapt to an external environment. Liu proposed that social adaptability includes social participation, learning, adaptability, and social support [39]. Studies have confirmed that social participation has a significant impact on cognitive impairment in older individuals. For example, Hsu found that participating in political groups reduced the risk of cognitive impairment in men [40]. Nie et al. found that cognitive function was significantly and positively correlated with social activity participation and the size of people’s families and friend networks, but not with their frequency of participation [41]. Jiang et al. studied the relationship between community social capital and cognitive function in terms of social support [42]. They found differences in the impact of community social capital indicators in different countries. For example, in India, Russia, and other countries, the level of trust in one’s neighbors was positively correlated with cognitive function. This did not appear to be the case in China, however, and in South Africa, social participation had close to zero effect. Feng et al. found that in China, social participation is an important health behavior that impacts the quality of life and cognitive function of older individuals with chronic diseases [43]. The above analysis indicates that the existing research on the classification indicators of social adaptability is relatively abundant, and many study conclusions have shown that social adaptability can affect the cognitive abilities of older individuals. Considering that social adaptability and air pollution exist concurrently, they may have different parallel effects on the cognitive abilities of older individuals; that is, there may be a regulatory effect between them. Therefore, this study also proposed a third hypothesis.

Hypothesis 3: Social adaptability has a significant positive effect on the cognitive abilities of older individuals and regulates the effect of air pollution.

### Data and methods

#### Data

We used the three-year follow-up survey data from the CHARLS database. Ethical approval for all CHARLS waves was provided by the institutional review board (IRB) of Peking University. The IRB approval number for the main household survey, including anthropometrics, was IRB00001052-11,015, and that of the biomarker collection was IRB00001052-11,014. During the fieldwork, each respondent who agreed to participate in the survey was asked to sign two copies of the informed consent form. One copy was kept in the CHARLS office and was also scanned and saved in PDF format. Four separate consents were obtained: one for the main fieldwork, one for the non-blood biomarkers, one for the collection of the blood samples, and one for the storage of blood for future analyses.

This study selected CHARLS survey data from 2015 and 2018. The CHARLS database mainly covers the basic health status surveys of people over 45 years of age in more than 20 regions in China and accurately reflects the current basic health level of the Chinese elderly population. Macro data, such as air quality, are based on meteorological statistics for various regions in China for each year (Air Quality Online Detection and Analysis Platform, https://www.aqistudy.cn/) and the monthly national air quality report published by the Ministry of Ecology and Environment of the People’s Republic of China. In this study, the annual average air quality and classification indicators of the survey area each year were selected to reflect the cognitive statuses of older individuals who had lived under a certain air quality for a long time. To avoid the error caused by the lack of samples, we choose the linear imputation method for processing the missing values. The linear imputation method is based on ID and year and generates a new corresponding variable through the ipolate and epolate commands. Using the two phases of CHARLS microsurvey data, combined with the relevant statistical air pollution data in each region (122 cities) in each year and through the relevant regional and community identification documents (IDs), we used Stata 15 to match the data and obtained 28,395 valid observations of data panels relevant to cognitive impairment in older individuals. Definitions of the core variables are listed in Table 1.

**Table 1:** Variable definitions

Variable	Definition
Cognitive	According to the concise community cognitive impairment screening scale (CSI-D). Due to the differences between the problems related to cognitive impairment in the CHARLS survey database in 2015 and 2018, when summing the relevant cognitive data, we defined 0 and 1 as having a cognitive impairment and having no cognitive impairment, respectively
AQI	Is based on the average value of 365 days in the survey year. For example, the regional AQI value in 2015 is based on the average value of AQI in the survey cities from January to December of 2015, and the regional AQI value in 2018 is also based on the same method
AQI_100day	Number of days with AQIs ≥ 100 in the survey year
AQI_150day	Number of days with AQIs ≥ 150 in the survey year
AQI_200day	Number of days with AQIs ≥ 200 in the survey year
AQI_300day	Number of days with AQIs ≥ 300 in the survey year
Fiscal expenditure	Regional annual fiscal expenditure level, 100 million yuan/year
Sunshine duration	Total annual sunshine hours in the region, h/year
Annual rainfall	Regional annual rainfall, mm/year
GDP per capita	Regional per capita GDP level
Population density	Regional population density
Average temperature	Regional annual average temperature, °C
Green coverage	Regional greening rate level
Education	The highest diploma obtained by respondents at the time of the survey
Sex	Men = 1; Women = 0
Type of residence	1 = Family housing, 2 = Nursing home, 3 = Hospital, 4 = Other
Health insurance	Individuals participating in any kind of basic insurance or commercial insurance, enjoying social assistance, and others = 1; no = 0
Marital status	Married = 1; never married, divorced, widowed = 0
Age	The actual age of the respondents at the time of the survey
Individual income	The total income level of the surveyed year, including wage income, pension, and other transfer payment income, yuan/year
Self-rated health	1 − 5 representing Very Good, Good, Fair, Poor, and Very Poor, respectively
Smoking	1 = yes; 2 = no
Drinking	1 = I have never had a drink; 2 = I used to drink less than once a month; 3 = I used to drink more than once a month
BADL	Basic ADLs are summed up according to six major problems of older individuals such as dressing, bathing, eating, getting up, getting out of bed, going to the toilet, and controlling urination and defecation. The corresponding options 1–4 are "no, I do not have any disability; I have a disability but can still do it; yes, I have a disability and need help; and I cannot do it". The total score is 6–24 points. The larger the value, the lower the basic self-care ability
IADL	The instrumental ADL is obtained according to 15 specific items concerning older individuals, such as housework, cooking, taking medicine, buying groceries, and financial management, and the corresponding options are the same as BADL. The total value is between 15 and 60. The larger the value, the worse the instrumental self-care ability
Social adaptability	According to social interaction, learning, adaptability, and social support

Note: According to the entropy weight method, the weights of social communication, learning and adaptability, and social support in social adaptability are 0.3159, 0.3407, and 0.3433 respectively

*AQI* Air quality index, *BADL* Basic activities of daily living, *CSI-D* Community screening instrument for dementia, *GDP* Gross domestic product, *IADL* Instrumental activities of basic living

Based on Table 1, we synchronously investigated the changes in the basic health indicators and macro-environmental indicators of older individuals in China logged in the two surveys and conducted a mean difference test. As shown in Table 2, from 2015 to 2018, the core statistical variables of cognition and AQI showed significant mean differences, indicating that over time variables related to air quality in older individuals changed, as did health-related statistical indicators. In 2018, 26.81% of the older individuals in the sample had no cognitive impairment, compared to 31.12% in 2015.

**Table 2:** Mean difference test of individual core variables over two surveys

Variables	G1 (2015)	Mean 1	G2 (2018)	Mean 2	Mean Diff
Cognition	10,436	0.3112	17,959	0.2681	0.0431^***^
ln(AQI)	10,436	4.4145	17,959	4.2516	0.1629^***^
Sex	10,436	0.4865	17,959	0.4795	0.007
Age	10,436	68.0093	17,959	60.9888	7.0205^***^
Type of residence	10,436	1.0271	17,959	1.0651	-0.0379^***^
Education	10,436	3.157	17,959	3.4048	-0.2478^***^
Marital status	10,436	0.7891	17,959	0.8415	-0.0524^***^
Smoking	10,436	1.9065	17,959	1.9132	-0.0067^***^
Drinking	10,436	2.4416	17,959	2.4065	0.0351^***^
Self-rated health	10,436	3.0031	17,959	2.9556	0.0475^***^
BADL	10,436	6.6369	17,959	4.8633	1.7736^***^
IADL	10,436	21.7244	17,959	20.1923	1.5321^***^
ln(SO2)	10,436	3.1741	17,959	2.6965	0.4776^***^
ln(NO2)	10,436	3.4472	17,959	3.5964	-0.1492^***^
ln(PM10)	10,436	4.4689	17,959	4.4013	0.0677^***^
ln(Fiscal expenditure)	10,436	5.9751	17,959	6.1635	-0.1884^***^
ln(Sunshine duration)	10,436	7.4527	17,959	7.527	-0.0743^***^
ln(Annual rainfall)	10,436	6.8097	17,959	6.7961	0.0136^**^
ln(Land area)	10,436	9.5865	17,959	9.6311	-0.0446^***^
Green coverage	10,436	39.7178	17,959	39.971	-0.2532^***^
Relative humidity	10,436	65.2091	17,959	65.1816	0.0275
ln(GDP_per_capita)	10,436	10.597	17,959	10.7862	-0.1892^***^
ln(Population density)	10,436	5.8143	17,959	5.7653	0.0490^***^
ln(Average temperature)	10,436	2.6995	17,959	2.671	0.0285^***^
ln(individual income)	10,436	1.8979	17,959	6.2005	-4.3026^***^
Social adaptability	10,436	0.2375	17,959	0.2917	-0.0543^***^
Health insurance	10,436	0.7811	17,959	0.9693	-0.1882^***^
AQI_100day	10,436	65.1306	17,959	56.9936	8.1370^***^
AQI_150day	10,436	17.9629	17,959	12.359	5.6040^***^
AQI_200day	10,436	12.5425	17,959	4.4807	8.0618^***^
AQI_300day	10,436	2.653	17,959	0.5846	2.0684^***^

Note: The above data were obtained by processing the initial sample data; the decrease in the sample size of the empirical analysis was mainly because the samples with missing values were eliminated in the empirical process of this study

*AQI* Air quality index, *BADL* Basic activities of daily living, *GDP* Gross national product, *IADL* Instrumental activities of basic living, *ln* Natural logarithm, *NO*_*2*_ Nitrogen dioxide, *PM*_*10*_ Particulate matter ≤ 10 µm, *SO*_*2*_ Sulfur dioxide

***p < 0.01

**p < 0.05

### Benchmark model

We first established a benchmark model to determine the health effects of air pollution. Cognitive impairment in older individuals was selected as the core proxy variable. For the core explanatory variable, the average AQI of the city in which the survey sample was conducted was selected as the proxy. The AQI value, therefore, represented the average daily value in a given survey year for a specific city. Yearly AQI values were based on the average value of 365 data points in the survey year. For example, the regional AQI value in 2015 was based on the average value of AQI in the survey cities from January to December 2015, and the regional AQI value in 2018 was also based on the same method. Based on these variable settings, we attempted to establish a model for estimating the cognitive effects of air pollution as follows:

1 $$Cognitive_{ijt} = \alpha_{0} + \alpha_{1} \ln AQI_{ijt} + \alpha_{2} X_{ijt} + \tau_{t} + \omega_{i} + \varepsilon_{ijt}$$

where $i$ represents individuals,$j$ represents groups, and $t$ represents time.$Cognition$ is the core explanatory variable of cognitive ability, which was obtained from the community screening instrument for dementia (CSI-D) used in the interviews. A larger value indicated a more serious cognitive impairment in older individuals. Since we used survey data from 2015 and 2018, to facilitate our analysis, we defined cognition as a dummy variable; namely, we defined all correct answers as 1, indicating no cognitive impairment, and those with one or more wrong answers as 0, indicating cognitive impairment. Specific definitions are shown in Table 1, and the natural logarithm ln(AQI) represents the logarithm of the air pollution value. Based on existing relevant research, we adopted the often-used proxy indicators for air pollution: API and AQI. API mainly includes three statistical indicators: SO_2_, NO_2,_ and PM_10_; AQI, meanwhile, adds another three pollutant indicators: fine particulate matter (PM_2.5_), ozone, and carbon monoxide (CO), giving it the advantage of comprehensiveness. Therefore, this study selected the annual average AQI value for a city during each survey year as a proxy variable. Unlike previous studies, this one also focused on the effects of pollution intensities and pollutants on cognitive impairment in older individuals.

Pollution intensity was quantified as the number of days of extreme air pollution per year. Days with AQI ≥ 100 indicate a light intensity of pollution and are recorded as AQI_100day; days with AQI ≥ 150 indicate medium pollution intensity and are recorded as AQI_150day; days with AQI ≥ 200 indicate the intensity of heavy pollution and are recorded as AQI_200day; days with AQI ≥ 300 indicate severe pollution intensity and are recorded as AQI_300day. Finally, the statistical indicators contained in API were mainly selected for classification tests of pollutant effects, while at the same time,$X_{ijt}$ Model (1) represents control variables, mainly including individual and regional characteristic variables, referring to the research of Bert and Stephen [44] and Kumar et al. [45]. To reduce the impact of other factors, we controlled for individual characteristic variables, such as age, sex, education, and health, as well as regionally fitted variables, such as regional per capita GDP and average rainfall in the model.$\tau$ and $\omega$ represent time and individual effects, respectively, and $\varepsilon$ represents random error terms. The definitions and statistical values of the relevant variables are presented in Tables 1 and 2, respectively. $\alpha_{0}$ is the constant term, $\alpha_{1}$ is the core coefficient of this study, and $\alpha_{2}$ is the correlation coefficient of the control variable.

Furthermore, because the social adaptability of older individuals has a great impact on cognitive ability [39], to ensure the robustness of the estimation results, we also controlled for social adaptability and the interaction between air pollution and social adaptability in this model. The test model is set as follows:

2 $$Cognitive_{ijt} = \alpha_{0} + \alpha_{1} \ln AQI_{ijt} + \alpha_{2} Social\_adapt_{ijt} + \alpha_{3} Interactive_{ijt} + \alpha_{4} X_{ijt} + \tau_{t} + \omega_{i} + \varepsilon_{ijt}$$

Model (2):$Social\_adapt_{ci}$ represents the social adaptability of older individuals, and $Interactive$ represents the interaction between social adaptability and air pollution. The other control variables were consistent with those in Model (1).$\alpha_{0}$ represents the constant term; the coefficients and symbols of $\alpha_{1}$, $\alpha_{2}$, and $\alpha_{3}$ are the focus of this study; and $\alpha_{4}$ represents the correlation coefficient of the control variable. $X_{ijt}$, $\tau_{t}$,$\omega_{i}$, and $\varepsilon_{ijt}$ are defined in the same manner as in Model (1).

### Two-stage instrumental variable model

Due to a selection bias associated with the air pollution samples and the omission of control variables, an endogeneity of the results may have occurred. Therefore, in the subsequent analysis, we conducted a test of sample-selective error by controlling for the samples after environmental migration and two-way fixed effects. In addition, we used an instrumental variable method for the endogenous treatment. A two-stage instrumental variable model was adopted for processing. Usually, the regional annual average wind speed significantly correlates with regional air pollution, and no direct correlation occurs between the regional annual average wind speed and cognitive impairment in older individuals. Thus, the regional annual average wind speed variable meets the exogenous assumption and basic requirements of the instrumental variables. Accordingly, the two-stage instrumental variable was set as follows:

3 $$\left\{ \begin{gathered} \ln AQI_{ijt} = \beta_{0} + \beta_{1} \ln wind\_speed_{ijt} + \beta_{2} X_{ijt} + \tau_{t} + \omega_{i} + \delta_{ijt} \hfill \\ Cognitive_{ijt} = \chi_{0} + \chi_{1} \ln AQI_{ijt} + \chi_{2} X_{ijt} + \tau_{t} + \omega_{i} + \varsigma_{ijt} \hfill \\ \end{gathered} \right.$$

The first stage of Model (3) is the ordinary least squares (OLS) regression of the instrumental variable ln(wind_speed) to ln(AQI). The fitted value of air pollution obtained at this stage was then used to test whether it was endogenous, which was mainly determined by the second stage of the Model (3). Subsequently, OLS regression was performed to obtain a consistent estimation. This process proceeded mainly through a two-stage least squares (2SLS) estimation. The definitions of $X_{ijt}$, $\tau_{t}$, and $\omega_{i}$ were the same as in Model (1), and that of $\varepsilon_{ijt}$ is the same as that of $\delta_{ijt}$ and $\varsigma_{ijt}$.

### Causal stepwise analysis model

To further analyze the internal mechanism behind the impact of air pollution on cognitive ability in older individuals, we used a causal step-by-step analysis. The specific model was set as follows:

4 $$Health_{ijt} = f_{0} + f_{1} \ln AQI_{ijt} + f_{2} X_{ijt} + \tau_{t} + \omega_{i} + \varepsilon_{ijt}$$

5 $$Cognitive_{ijt} = x_{0} + x_{1} \ln AQI_{ijt} + x_{2} Health_{ijt} + x_{3} X_{ijt} + \tau_{t} + \omega_{i} + \varepsilon_{ijt}$$

In Model (4), $Health$ is the health index for older individuals. In this study, basic activities of daily living (BADL), instrumental activities of daily living (IADL), and self-rated health were used as agents. This study focused on the coincidence of the coefficients $f_{1}$ and $x_{2}$ in Models (4) and (5). If both $f_{1}$ and $x_{2}$ were significant, it indicated that there was a complete mediation effect, whereas if $f_{1}$ and $x_{2}$ were partially significant it indicated that there was only a partial mediation effect. The definitions of $\tau_{t}$, $\omega_{i}$, and $\varepsilon_{ijt}$ are the same as those presented in Model (1).

## Results

### Benchmark test

Following the model design and data sets, we first started with a benchmark model to empirically test the impact of air pollution on cognition in older individuals. The empirical test results, which include the four models, are shown in Table 3. The tests were performed using Stata 15. Model (1) did not control for individual health variables. The results showed that AQI had a significant negative impact on cognition in older individuals, indicating that when the regional AQI value is higher, the cognitive impairment of older individuals will likely be more serious, with an odds ratio (OR) of 1.4633 (95% CI: 1.20899–1.77116). This indicated that air pollution significantly increases the cognitive impairment of older individuals. Model (2) was a test of the individual health variables. The results showed that the regional AQI values also had significant negative effects on cognitive impairment. Models (3) and (4) show the results of the tests after adding the variables of social adaptability and interaction items to the first three models, respectively. The results of Model (3) show that regional AQI values have significant negative impacts on the cognitive abilities of older individuals and that social adaptability has a significant positive impact, with ORs of 1.3579 (95% CI: 1.12889–1.63357) and 1.1234 (95% CI: 1.01316–1.26571), respectively. Compared to older individuals with low social adaptability, the odds of good cognitive ability in older individuals with high social adaptability increased 1.1234 times. The results of Model (4) show that the interaction between social adaptability and regional air quality has significant positive effect; that is to say, when regional air quality is at a certain level, enhancing the social adaptability of older individuals will improve the cognitive impairment caused by air pollution.

**Table 3:** Benchmark regression test results

Variables	Explained variable: Cognitive ability			
	**(1)**	**(2)**	**(3)**	**(4)**
**ln(AQI)**	-0.3807^***^ (0.0974)	-0.3085^***^ (0.0943)	-0.3060^***^ (0.0943)	-0.3413^***^ (0.0945)
**Social adaptability**	-	-	0.1244^**^ (0.0568)	0.1488^***^ (0.0569)
**ln(AQI) × ** **Social adaptability**	-	-	-	0.0150^***^ (0.0027)
**Sex**	0.7252^***^ (0.0545)	0.6732^***^ (0.0524)	0.6816^***^ (0.0525)	0.6717^***^ (0.0524)
**Age**	0.0350^***^ (0.0035)	0.0428^***^ (0.0036)	0.0429^***^ (0.0036)	0.0433^***^ (0.0035)
**Type of residence**	-0.3484^***^ (0.0594)	-0.3494^***^ (0.0572)	-0.3480^***^ (0.0572)	-0.3466^***^ (0.0570)
**Education**	0.2313^***^ (0.0137)	0.1875^***^ (0.0135)	0.1819^***^ (0.0137)	0.1859^***^ (0.0137)
**Marital status**	0.2104^***^ (0.0633)	0.0538 (0.0616)	0.0544 (0.0616)	0.0542 (0.0614)
**Smoking**	0.2083^**^ (0.1005)	0.1976^**^ (0.0978)	0.1989^**^ (0.0977)	0.2012^**^ (0.0975)
**Drinking**	0.0189 (0.0275)	0.0619^**^ (0.0267)	0.0664^**^ (0.0268)	0.0644^**^ (0.0267)
**ln(individual income)**	0.0914^***^ (0.0051)	0.0986^***^ (0.0051)	0.0981^***^ (0.0051)	0.0983^***^ (0.0050)
**Health insurance**	0.1080 (0.0714)	0.1275^*^ (0.0701)	0.1260^*^ (0.0701)	0.1289^*^ (0.0699)
**ln(Fiscal expenditure)**	-0.3040^***^ (0.0609)	-0.2801^***^ (0.0588)	-0.2810^***^ (0.0588)	-0.2841^***^ (0.0586)
**ln(Land area)**	0.4669^***^ (0.0837)	0.4274^***^ (0.0807)	0.4286^***^ (0.0806)	0.4342^***^ (0.0804)
**Green coverage**	0.0123^***^ (0.0034)	0.0111^***^ (0.0033)	0.0110^***^ (0.0033)	0.0113^***^ (0.0033)
**ln(Sunshine duration)**	0.1240 (0.1161)	0.2278^**^ (0.1124)	0.2350^**^ (0.1124)	0.2313^**^ (0.1120)
**ln(Annual rainfall)**	0.1698^**^ (0.0796)	0.1282^*^ (0.0769)	0.1288^*^ (0.0768)	0.1325^*^ (0.0766)
**Relative humidity**	0.0013 (0.0040)	0.0006 (0.0039)	0.0007 (0.0039)	0.0009 (0.0039)
**ln(Average temperature)**	-0.8715^***^ (0.1153)	-0.8769^***^ (0.1112)	-0.8746^***^ (0.1111)	-0.8906^***^ (0.1108)
**ln(Population density)**	0.4234^***^ (0.0661)	0.4056^***^ (0.0639)	0.4067^***^ (0.0639)	0.4092^***^ (0.0636)
**ln(GDP_per_capita)**	0.4642^***^ (0.0513)	0.4245^***^ (0.0495)	0.4230^***^ (0.0495)	0.4225^***^ (0.0493)
**BADL**		0.0911^***^ (0.0080)	0.0910^***^ (0.0080)	0.0904^***^ (0.0080)
**IADL**		-0.0809^***^ (0.0038)	-0.0800^***^ (0.0038)	-0.0795^***^ (0.0038)
**Self-rated health**		0.2024^***^ (0.0248)	0.2038^***^ (0.0248)	0.2017^***^ (0.0247)
**Constant**	-17.7997^***^ (1.7489)	-16.8367^***^ (1.6910)	-16.9451^***^ (1.6908)	-17.1619^***^ (1.6867)
**Log-likelihood**	-15,408.874	-15,147.273	-15,144.881	-15,128.786
**LR test**	1080.33^***^	860.40^***^	855.67^***^	840.27^***^
**Observations**	28,395	28,395	28,395	28,395
**Groups**	19,655	19,655	19,655	19,655

Note: Standard errors are in parentheses. ^***^p < 0.01, ^**^p < 0.05, and ^*^p < 0.1. OR values are not reported in the table

*AQI* Air quality index, *BADL* Basic activities of daily living, *GDP* Gross national product, *IADL* Instrumental activities of basic living, *ln* Natural logarithm, *LR* Likelihood ratio, *OR* Odds ratio

### The effects of air pollution intensity and pollutants

Based on our benchmark analysis, we further investigated the effects of pollution intensity and pollutants on cognitive impairment in older individuals. The results are presented in Table 4. In terms of the impact of the pollution intensity, we used the cumulative number of days when the regional annual AQI value was greater than 100, 150, 200, and 300 as the extreme statistical limits of different pollution intensities. Models 1–4 are the estimations of four levels of pollution intensity. From Table 4, it can be seen that with increases in extreme pollution days, cognitive abilities in older individuals were significantly impacted, mainly showing a negative decreasing effect. Moreover, only the cumulative number of days when the regional annual AQI value was greater than 200 or more will have a significant effect.When the AQI is below 200, the pollution intensity has no significant impact on the cognitive abilities of older individuals.The ORs of AQI_200day, and AQI_300day were 1.0032 (95% CI: 1.00058–1.00572), 1.0159 (95% CI: 1.00447–1.02750), respectively. The test results showed that the intensity of air pollution has a significant impact on cognitive ability in older individuals, but that as pollution intensity increases, this impact remains stable. Therefore, this shows that the second half of Hypothesis 2 is not consistent with empirical evidence; that is, an increase in air pollution intensity does not significantly enhance its impact on the cognitive abilities of older individuals.

**Table 4:** Air pollution intensity and cognitive impairment of the older individuals

Variables	Explained variable: Cognitive ability				
	**(1)**	**(2)**	**(3)**	**(4)**	**(5)**
**AQI_100day**	0.9995 (0.0007)				
**AQI_150day**		1.0012 (0.0017)			
**AQI_200day**			1.0032^**^ (0.0013)		
**AQI_300day**				1.0159^***^ (0.0058)	
**ln(SO2)**					1.3802^***^(0.0474)
**ln(NO**_**2**_**)**					1.0539(0.0841)
**ln(PM**_**10**_**)**					0.9267(0.0843)
**Constant**	1.59e-07^***^	-15.3509^***^	-15.1370^***^	-14.8540^***^	-14.4313^***^
	(2.64e-07)	(1.6532)	(1.6499)	(1.6570)	(1.6933)
**Log-likelihood**	-15,153.804	-15,153.815	-15,151.258	-15,150.401	-15,128.976
**LR test**	867.84^***^	868.18^***^	865.66^***^	859.70^***^	838.40^***^
**Observations**	28,395	28,395	28,395	28,395	28,395
**Groups**	19,655	19,655	19,655	19,655	19,655

Note: Standard errors in parentheses, ^***^p < 0.01, ^**^p < 0.05, ^*^p < 0.1. The OR values are reported in the table, the impact coefficient symbols of air pollution variables are all negative, and the results of control variables, such as environmental characteristics and individual characteristics, are not listed

*AQI* Air quality index, *ln* Natural logarithm, *LR* Likelihood ratio, *NO*_*2*_ Nitrogen dioxide, *PM*_*10*_ Particulate matter of 10 µm or less, *SO*_*2*_ Sulfur dioxide

In addition, based on the impact of air pollution intensity, we further investigated the impact of different pollutants on older individuals, the results of which are shown in Table 4 and Model (5). These results show that ln(SO_2_) for different pollutants have a significant negative impact on the cognitive abilities of older individuals, with ORs of 1.3802 (95% CI: 1.25779–1.51451), indicating that SO_2_ has a significant impact on the cognitive ability of older individuals but that NO_2_ and PM_10_ does not. This is consistent with the conclusions of previous studies [8]. For example, several studies have revealed that if people are exposed to SO_2_ for a long time, the upper respiratory tract is stimulated, bronchial smooth muscle is contracted, respiratory resistance is increased, and respiratory functions decrease. SO_2_ increases and thickens the secretion of respiratory mucosa hinders the movement of cilia, weakens immune function, reduces the resistance of the respiratory tract, induces inflammation to varying degrees, and leads to mental retardation [46–49].

### Group heterogeneity analysis

To investigate the heterogeneity of the effects of air pollution on cognitive impairment in the different groups, we examined group heterogeneity from two perspectives: individual characteristics and regional characteristics. The individual characteristics were mainly investigated from three perspectives: age, sex, and education. Regional characteristics were mainly investigated from the perspective of the regional economic level. The results are shown in Tables 5 and 6. In terms of individual characteristics, for the age-group differences, we divided the data into older individuals < 70 years and ≥ 70 years. In Table 5, the results of Models (1) and (2) tested by the sample from the perspective of age show that the regional AQI value does have a significant impact on cognitive ability in older individuals of all aged. Social adaptability was found to have a significant positive effect on those aged ≥ 70 years,but does not have a significant impact on cognitive ability in older individuals of aged < 70 years. However, the interaction between AQI and social adaptability only had a significant positive effect on the cognitive ability of older individuals aged < 70 years, showing no effect in the > 70 group. In terms of sex differences, the results of Models (3) and (4) in Table 5 show that the regional AQI value does have a significant negative impact on the cognitive abilities of older individuals of both sexes. Social adaptability has no significant impact on cognitive ability in older men but does have a significant positive impact on older women. The interaction between AQI and social adaptability has a significant impact on the cognitive abilities of older individuals of both sexes. Third, in terms of education, Models (5) and (6) in Table 5 compare the group differences in terms of the level of education, taking elementary school as the “boundary,” elementary school and below being a “low level of education,” and above elementary school indicating a “high level of education.” The results show that the regional AQI value has a significant negative effect on cognitive ability in older individuals with low education levels but not in those with high education levels. Social adaptability was found to have a significant positive effect on the cognitive ability of older individuals irrespective of educational level in both groups, but its interaction with AQI had also significant effect on the cognitive ability of older individuals in both groups. These results show that, in terms of individual characteristics, the influence of regional AQI on cognitive ability in older individuals shows obvious group heterogeneity in terms of age, sex, and education level. However, social adaptability and the interaction between AQI and social adaptability showed group heterogeneity only with regard to age.

**Table 5:** Air pollution and cognitive impairment of older individuals under individual differences

Variable	Explained variable: Cognitive ability					
	**Age**		**Sex**		**Education**	
	**Under 70 (1)**	**Over 70 (2)**	**Men (3)**	**Women (4)**	**High (5)**	**Low (6)**
**ln(AQI)**	-0.2422^**^	-0.8348^***^	-0.2694^**^	-0.4076^***^	-0.0110	-0.2798^***^
	(0.0999)	(0.2360)	(0.1211)	(0.1511)	(0.2168)	(0.0995)
**Social adaptability**	0.0648	1.1023^***^	-0.0449	0.3814^***^	-0.2863^***^	0.6202^***^
	(0.0584)	(0.1855)	(0.0735)	(0.0902)	(0.0989)	(0.0683)
**AQI × Social adaptability**	0.0140^***^	-0.0006	0.0155^***^	0.0147^***^	0.0092^*^	0.0137^***^
	(0.0028)	(0.0067)	(0.0034)	(0.0043)	(0.0053)	(0.0030)
**Constant**	-23.8168^***^	-9.0764^**^	-14.4226^***^	-17.5948^***^	-18.4351^***^	-16.2400^***^
	(1.8179)	(4.3494)	(2.1652)	(2.6986)	(3.6931)	(1.7997)
**Log-likelihood**	-12,746.842	-2147.3222	-8169.1416	-6844.8511	-3840.0101	-11,105.618
**Observations**	23,790	4,605	13,707	14,688	6,486	21,909
**Groups**	15,638	4,017	9,503	10,152	6,179	13,476

Note: Standard errors in parentheses, ^***^p < 0.01, ^**^p < 0.05, ^*^p < 0.1. The results for the control variables, such as environmental and individual characteristics, are not listed

*AQI* Air quality index

**Table 6:** Air pollution and cognitive impairment of older individuals in terms of regional economic differences

Variable	Explained variable: Cognitive ability	
	**Developed regions (1)**	**Underdeveloped regions (2)**
**ln(AQI)**	-0.0353 (0.2085)	0.3976^***^ (0.1119)
**Social adaptability**	0.2675^**^(0.1051)	0.1102 (0.0672)
**AQI × Social adaptability**	0.0107^**^ (0.0051)	0.0165^***^ (0.0031)
**Constant**	-7.1255 (6.4059)	-19.9252^***^ (1.9083)
**Log-likelihood**	-4033.6754	-11,116.818
**Observations**	6,923	21,472
**Groups**	5,330	14,325

Note: Standard errors in parentheses, ^***^p < 0.01, ^**^p < 0.05, ^*^p < 0.1. The results for the control variables, such as environmental and individual characteristics, are not listed

*AQI* Air quality index

In terms of regional characteristics based on the regional economic development level (mainly GDP), we took the regional GDP mean value in the survey sample as the dividing point. Those above the mean value were defined as developed regions, and those equal to or below the mean value were defined as underdeveloped regions to investigate the heterogeneity of air pollution impacts under the difference between developed and underdeveloped regions. The results of Models (1) and (2) in Table 6 show that the regional AQI value has a negative effect on cognitive ability in older individuals in underdeveloped areas but has no significant effect on cognitive ability in older individuals in developed areas. However, social adaptability only has a positive effect on cognitive ability in older individuals in developed areas. The interaction of AQI and social adaptability does have a significant heterogeneity in terms of effects on cognitive ability in older individuals, in both underdeveloped and developed regions. These results show that air pollution has significant effects depending on regional economic development heterogeneity on cognitive impairment in older individuals and that its impact is more significant in underdeveloped areas.

### Further analysis

#### Robustness test

There may have been estimation errors in these tests. Firstly, the estimation results are likely to be unstable owing to errors in the sample selection. Secondly, owing to the endogeneity of the air pollution variable, the research conclusions may be unreliable. Therefore, we first controlled the samples to reduce the estimation error caused by the sample selection error. In this step, we mainly reduced the impact of environmental migration by excluding samples from people who had relocated once, according to population household registration, thereby increasing the reliability of our conclusions. For example, concerning serious air pollution, household registration relocation appears to avoid some of the health damage caused by air pollution. If such samples are included in estimations, bias in the results may arise. Table 7, Model (1), shows the estimated results after controlling for population migration. The results showed that regional AQI values still had a significant negative impact on cognitive impairment in older individuals (OR: 1.2699; 95% CI: 1.04709–1.54015), indicating that the benchmark test results were robust.

**Table 7:** Robustness test

Variables	Explained variable: Cognitive ability			
	**Control population migration (1)**	**Two-way fixed effect (2)**	**Instrumental variable method (3)**	
			**First stage (ln[AQI])**	**Second stage**
**ln(AQI)/ln(Wind_speed)**	1.2699^**^	1.3180^***^	-0.0475^***^	-0.6016^***^
	(0.1250)	(0.1244)	(0.0043)	(0.1817)
**Constant**	8.73e-08^***^	6.12e-07^***^	2.9232^***^	0.5906
	(1.54e-07)	(1.07e-06)	(0.0908)	(0.5648)
**Province/Year**	-	YES	-	-
**Log-likelihood**	-13,544.339	-15,135.839	-	-
**LR test/R**^**2**^	663.78^***^	859.95^***^	0.3146	0.4514
**First stage F value**	-	-	612.96	-
**Observations**	28,395	28,395	28,395	28,395
**Groups**	19,655	19,655	-	-

Note: Standard errors in parentheses, ^***^p < 0.01, ^**^p < 0.05, ^*^p < 0.1. The results of control variables such as environmental characteristics and individual characteristics are not listed

*AQI* Air quality index, *LR* Likelihood ratio

Furthermore, although the variables of family and individual characteristics, as well as regional environmental characteristics, were all controlled for in the above analysis, there may still have been missing variables that introduced bias in the estimation. To deal with this endogeneity problem, we first selected a two-way fixed-effects model to fix the endogeneity problem caused by missing variables. Referring to a previous study by Liu and Hu [8], using classified variables as continuous variables and a linear two-way fixed-effects model estimation are possible options. We also adopted a two-way panel fixed-effects method. Two-way fixation was achieved by controlling for time and regional effects, and variables such as cognitive impairment were viewed as continuous variables. The results are shown in Model (2) in Table 7, and demonstrate that after the implementation of the two-way fixed effect model, the regional AQI value still had a significant negative impact on cognitive impairment in older individuals, with an OR of 1.3180 (95% CI: 1.09547–1.58592), which is close to the results of the benchmark model, indicating that the findings of benchmark tests were robust.

Finally, to avoid the influence of endogeneity problems, we chose the instrumental variable method. For the selection of instrumental variables, based on the instrumental variable method used by Liu [39], the regional annual average wind speed was selected as the instrumental variable, and the IV model was used for the estimation. Part 3.2 of the article determined that the regional average wind speed met two basic conditions for the selection of instrumental variables. In addition, the first-stage results of the two-stage instrumental variable method in Model (3) in Table 7 show that ln(wind_speed) has a significant negative effect on ln(AQI). The F value in the first stage is 612.96, which is significantly greater than 10, indicating that the selection of instrumental variables is effective. The results of the second stage of Model (3) in Table 7 show that air pollution still has a significant negative impact on cognitive ability in older individuals, with an impact coefficient of -0.6016 (95% CI: [-0.95774 to -0.24539]). In other words, when the regional annual average AQI increases by one unit, cognitive ability in older individuals is reduced by 60.16%. Endogenous treatment results confirmed the robustness of these benchmark test results.

### Sensitivity analysis

Since the benchmark model select the linear imputation method for processing missing values, this may have also led to an error in our estimation results. To avoid the error caused by linear imputation methods, we choose the mean imputation method and deleted-missing-value method for processing the sensitivity. The mean imputation method used the annual survey mean (sample mean) of each variable to replace all missing values; deleted-missing-value method means that we directly deleted the missing values when selecting basic data. The inspection results are shown in Table 8, where it can be seen that the core variable AQI still had a significant impact after the missing samples were processed by the mean imputation or deleted-missing-value method, indicating that the benchmark test results were robust. The linear imputation methods used in this paper therefore likely represents the overall sample distribution to some extent.

**Table 8:** Sensitivity test results

Variables	Mean imputation (1)		Linear imputation (2)	
	**coefficient**	**SE**	**coefficient**	**SE**
**ln(AQI)**	-0.3413^***^	(0.0945)	-0.5819^**^	(0.2501)
**Social adaptability**	0.1488^***^	(0.0569)	0.8904^***^	(0.1821)
**ln(AQI) × Social adaptability**	0.0150^***^	(0.0027)	0.0037	(0.0073)
**Sex**	0.6717^***^	(0.0524)	0.3348^**^	(0.1641)
**Age**	0.0433^***^	(0.0035)	-0.0140	(0.0098)
**Type of residence**	-0.3466^***^	(0.0570)	-0.2556	(0.1876)
**Education**	0.1859^***^	(0.0137)	0.6355^***^	(0.0638)
**Marital status**	0.0542	(0.0614)	0.2074	(0.1462)
**Smoking**	0.2012^**^	(0.0975)	-0.1120	(0.2309)
**Drinking**	0.0644^**^	(0.0267)	0.0426	(0.0830)
**ln(individual income)**	0.0983^***^	(0.0050)	0.0956^***^	(0.0158)
**Health insurance**	0.1289^*^	(0.0699)	0.5119^***^	(0.1599)
**ln(Fiscal expenditure)**	-0.2841^***^	(0.0586)	-0.0554	(0.1876)
**ln(Land area)**	0.4342^***^	(0.0804)	0.4051^*^	(0.2342)
**Green coverage**	0.0113^***^	(0.0033)	0.0026	(0.0079)
**ln(Sunshine duration)**	0.2313^***^	(0.1120)	0.9806^***^	(0.2856)
**ln(Annual rainfall)**	0.1325^*^	(0.0766)	-0.2475	(0.1812)
**Relative humidity**	0.0009	(0.0039)	0.0038	(0.0094)
**ln(Average temperature)**	-0.8906^***^	(0.1108)	-0.9104^***^	(0.3051)
**ln(Population density)**	0.4092^***^	(0.0636)	0.4465^**^	(0.1969)
**ln(GDP_per_capita)**	0.4225^***^	(0.0493)	0.6106^***^	(0.1468)
**BADL**	0.0904^***^	(0.0080)	0.0400^*^	(0.0230)
**IADL**	-0.0795^***^	(0.0038)	-0.0920^***^	(0.0121)
**Self-rated health**	0.2017^***^	(0.0247)	0.0108	(0.0614)
**Constant**	-17.1619^***^	(1.6867)	-14.2992^***^	(4.594)
**LR test**	840.27^***^		26.40^***^	
**Log-likelihood**	-15,128.786		-1988.3851	
**Observations**	28,395		3,750	
**Groups**	19,655		3,347	

Note: Standard errors in parentheses, ^***^p < 0.01, ^**^p < 0.05, ^*^p < 0.1. The results for the control variables, such as environmental and individual characteristics, are not listed

*AQI* Air quality index, *BADL* Basic activities of daily living, *GDP* Gross national product, *IADL* Instrumental activities of basic living, *ln* Natural logarithm, *LR* Likelihood ratio test

### Influence mechanism test

A previous study analyzed the direct effect of air pollution on cognitive ability in older individuals, but the specific impact mechanism for this has not yet been studied. Therefore, this section explores the reasons for the impact of air pollution on cognitive ability in older individuals, based on a previous article [8, 18, 21]. To investigate the mechanism behind the phenomenon, we used causal step-by-step analysis to investigate the intermediary impact of air pollution on cognitive ability in older individuals. Table 9 presents the results. Models (1) to (3) are the results of the first stage, where the impact of regional AQI on the health level of older individuals was investigated. The results revealed that regional AQI has a significant negative impact on the BADL, IADL and self-rated health of older individuals, with coefficients of 0.5715,-1.6543 and -0.3452, respectively. This indicates that when the regional AQI increases by one unit, the BADL of older individuals decrease by 0.5715,the IADL and self-rated health of older individuals decrease by 1.6543 and 0.3452, respectively. In addition, Model (4) in Table 8 shows that BADL, IADL and self-rated health, as proxy indicators of the health level of older individuals, had a significant effect on cognitive ability. Combined with the test results of Models (1–3), we found that in terms of the intermediary effect on health, air pollution mainly affects individual BADLs, IADLs and self-rated health, and then transmits their impact to cognitive ability. These results revealed a mediating effect in the transmission mechanism of air pollution affecting cognitive ability in older individuals, which occurs mainly through the influence of BADL, IADL and self-rated health. They also confirm that Hypothesis 1 is correct: air pollution significantly affects the health of older individuals. Direct and indirect health-impact mechanisms also exist.

**Table 9:** The mechanism of how air pollution affects cognitive impairment in older individuals

Variables	BADL	IADL	Self-rated health	Cognitive
	**(1)**	**(2)**	**(3)**	**(4)**
**ln(AQI)**	0.5715^***^ (0.1870)	-1.6543^***^ (0.4038)	-0.3452^***^ (0.0597)	-
**BADL**	-	-	-	0.0914^***^ (0.0080)
**IADL**	-	-	-	-0.0813^***^ (0.0038)
**Self-rated health**	-	-	-	0.2031^***^ (0.0248)
**Constant**	15.2340^***^ (3.3906)	13.6172^*^ (7.3207)	-	-15.4733^***^ (1.6450)
**F test/LR test**	2.48^***^	3.10^***^	492.82^***^	868.43^***^
**R**^**2**^**/Log-likelihood**	0.0212	0.0831	-44,140.577	-15,154.091
**Observations**	28,395	28,395	28,395	28,395
**Groups**	19,655	19,655	19,655	19,655

Note: Standard errors in parentheses, ^***^p < 0.01, ^**^p < 0.05, ^*^p < 0.1. The results for the control variables, such as environmental and individual characteristics, are not listed

*AQI* Air quality index, *BADL* Basic activities of daily living, *GDP,* Gross national product; *IADL*, Instrumental activities of basic living, *ln* Natural logarithm, *LR* Likelihood ratio test

## Discussion

To our knowledge, this study is an extension of existing research on air pollution and cognitive impairment, such as that of Thompson et al. [50], Delgado–Saborit et al. [51], Calderón–Garcidueñas et al. [52], and Luo et al. [53]. Its core purpose was to test the intensities of air pollution and pollutants’ effects when introduced into the model and simultaneously examine the impact of social adaptability and air pollution on cognitive impairment in the elderly, to further enrich existing research perspectives and conclusions. Extensive research has been conducted on the impact of air pollution on the health of residents. For example, Liu [39] discussed the impact of air pollution on the health and medical consumption of older individuals from a multidimensional perspective, and several studies have shown that air pollution has a direct effect on the health of individuals, especially older individuals [16–18]. Results regarding the roles of individual mental health, ADL, self-rated health assessment, and other health agent indicators are also abundant [19–22]. In combining the conclusions of existing studies on the relationship between air pollution and individual health effects, we found that health damage is only a direct result and that it can lead to further indirect results such as individual cognitive function damage [23–28]. Liu and other mathematicians have further investigated the health impact of pollution on older individuals from the perspective of social adaptability [39–43]. Based on their research, this study attempted to explore the direct and indirect impact of air pollution on cognitive impairment in older individuals.

Firstly, the results of this study show that air pollution has a significant impact on cognitive impairment in older individuals, and this effect significantly increased after controlling for environmental migration, regional effects, and annual average wind speed. This, therefore, confirmed our Hypothesis 2—that air pollution can directly affect cognitive ability in older individuals. This conclusion is consistent with that of Jennifer et al. and Lindsay et al. [31–38]. In addition, the results of this study also show that different air pollution intensities have significant impacts on cognitive impairment in older individuals, with ORs that are relatively close (approximately 1.0). In this study, the number of days of extreme pollution in a year was selected as the proxy variable for air pollution intensity, where a range of AQI (AQI levels > 100, > 150, > 200, and > 300) was used as the criterion to identify extreme air pollution. We found that the pollution intensities had significant effects on cognitive impairment in older individuals when the AQI value is greater than 200 and that their OR values were similar. We, therefore, showed that air pollution had a peak effect on cognitive impairment in older individuals. When a certain pollution intensity was reached, the cognitive impairment of older individuals did not change with cumulative increases in pollution intensity, and the peak value of this pollution intensity was > 200. Hence, the second half of our Hypothesis 2 was not found to be consistent with empirical evidence; that is, increases in air pollution intensity significantly enhance its impact on cognitive ability in older individuals. The effect of increasing the cumulative number of days on cognitive ability in older individuals is relatively stable, which further suggests that air pollution has only a limited impact on cognitive impairment in older individuals even when the density of air pollution is extremely high. This study not only confirmed existing research conclusions in the literature but also expanded on the established impact of air pollution intensity on cognitive impairment.

Secondly, this study confirmed that the impact of air pollution on cognitive impairment in older individuals includes both direct and indirect effects. Thus, our Hypothesis 1 was confirmed. Consistent with the research conclusions of Stineman et al. [24], Corrêa et al. [25], and Kato [26], when ADL and self-rated health assessments were used as health proxy indicators, air pollution significantly affected ADL (significant impact on BALD and IADL) and the self-rated health assessments of older individuals. However, the ADL index and self-rated health assessment had a significant impact on cognitive function in older individuals. This result shows that air pollution affects the ADL and self-rated health assessment of older individuals, and its indirect effect is significant. Thus, the impact of air pollution on cognitive function in older individuals has indirect effects. Our Hypothesis 1 was therefore partially correct.

Thirdly, we also confirmed Hypothesis 3, which stated that social adaptability has a significant positive effect on cognitive ability in older individuals and that the interaction between social adaptability and regional air quality also has significant effect. By this, we mean that when the regional air quality is at a certain level, enhancing the social adaptability of older individuals will improve the cognitive impairment they have experienced caused by air pollution. The results of this study not only confirmed the first part of Hypothesis 3, but also confirmed the effect of social adaptation on cognitive ability in older individuals. An important contribution of this study is the parallel addition of social adaptability and air pollution to the model at the same time, to investigate the effects of both social and natural environmental factors. Traditional research on individual cognitive ability has focused on factors such as individual and family environments, ignoring the joint role of social and natural environments. Compared to the studies of Hsu [40], Nie et al. [41], Jiang et al. [42], and Feng et al. [43], this study, based on social participation as the main indicator, combined with the research of Liu [39], constructs a social adaptability index that not only examines the impact of social adaptability on cognitive impairment in older individuals but also demonstrates the joint effect of social adaptability combined with air pollution. Therefore, for the treatment of cognitive impairment in older individuals, we should not only investigate the characteristics of an individual’s physiology, family, and living environment but also explore the perspective of the natural environment, particularly air pollution. Based on the findings of existing research, air pollution affects outdoor activities, reduces social participation, and leads to more staying at home and less exercise, thus leading to the development of chronic diseases and even cognitive decline [42, 54–57].

Finally, in terms of the heterogeneity of different groups, this study indicates obvious group differences in the impact of air pollution on cognitive impairment in older individuals due to age, sex, education, and regional economic development. More specifically, individuals with lower education, and those in economically underdeveloped areas were significantly more affected by air pollution. Therefore, to optimize treatment measures for cognitive impairment, we should also consider policies preferentially targeting older individuals with lower education levels, and those residing in underdeveloped areas. Through such an approach, the pertinence and effectiveness of treatment plans can hopefully be improved.

The highlights of this study are that it not only investigated the impact of air pollution but also synchronously investigated the impact of different pollution intensities and pollutants on cognitive impairment in older individuals. This study also tested robustness by controlling for migration behavior using both two-way fixed effects and an instrumental variable method to ensure the reliability of the results. Based on age, sex, education, and regional economic development level, this study also investigated the group heterogeneity of the impact of air pollution on cognitive impairment in older individuals. Thus, the possibility of developing more reliable policy interventions for cognitive impairment was explored. This study confirms that air pollution causes cognitive impairment in older individuals. At the same time, our conclusion also shows that there is a peak value for the effects of air pollution on cognitive impairment in older individuals. As air pollution increases, the impact on the cognitive impairment of older individuals increases to a specific value, after which it does not fluctuate greatly and holds a relatively stable coefficient value.

However, some limitations of this study suggest directions for future research. (1) Air pollution has a significant negative effect on cognitive ability in older individuals, and its effect has a peak value. However, because of the limited availability of the research data, the exact peak value was not identified in this study, which is the main limitation of this study. Future research should combine survey and statistical data to address this shortcoming. (2) This study mainly focused on outdoor air pollution; however, there are differences between indoor and outdoor air pollution. Therefore, to effectively improve the reliability of our conclusions, we should also investigate the impact of indoor air pollution on cognitive impairment in older individuals. (3) Due to the period, this study only selected survey data from 2015 and 2018. In reality, the impact period of air pollution is much greater, over which short-term extreme fluctuation impacts are less likely. Therefore, another way to improve the reliability of our conclusions might be to attempt to track long-term panel data in the future for a more accurate group survey and analysis.

This study considered the cognitive abilities of older individuals, examined the impact of air pollution, and drew corresponding research conclusions. Based on the conclusions of this study, we present the following recommendations: First, air pollution has always been an inevitable problem in the economic development of all countries in the world. In China, a large developing country, local governments and management departments should also respond to the call of the central government to increase environmental governance, change modes of economic growth, and establish a green development path to reduce potential social governance costs, such as social healthcare costs, caused by extensive development. Second, in aiming to reduce negative impacts on cognitive ability in older individuals, relevant governance is mostly focused on the medical field, but the natural and social environments also have important impacts. Therefore, based on reasonable medical governance, we should strengthen the environmental prevention effort, focusing on interventions from the two important perspectives of air pollution and social integration, to improve cognitive function in older individuals or at least delay its decline. Third, considering the reality of the increasing medical expenses of older individuals and the increasing number of disabled older individuals, it is of great practical significance to intervene from an environmental perspective to improve overall health in this demographic. Concurrently, considering the heterogeneity of older individuals in terms of age, sex, education level, and other individual and regional characteristics such as economic development, effective social policy interventions need to be implemented differently for older individuals with different characteristics. Relevant policy-making should consider the current situation and common characteristics of cognitive ability in the older population, particularly in females, older individuals with low education levels, and those in economically developed areas, to improve the accuracy and effectiveness of policy intervention.

## Conclusions

The main conclusions of this study are as follows: First, air pollution has a negative impact on cognitive impairment in older individuals. A one-unit increase in the annual average AQI of a country or region increases the cognitive impairment of older individuals by 1.4633 times. Second, different pollution intensities also have significant negative impacts on cognitive impairment in older individuals, such as SO_2_ at different levels, for example. Third, social adaptability has a significant impact on cognitive impairment in older individuals, but before this study, it had not been adjusted in terms of describing the decline in cognitive function seen in older individuals caused by air pollution. Moreover, we have confirmed that the interaction between social adaptability and air pollution has an impact on the cognitive ability of older individuals.Fourth, an obvious group heterogeneity was observed in terms of age, sex, education, and regional economic development level when considering the impact of air pollution on cognitive impairment in older individuals. Air pollution was found to have a more significant impact on older individuals, those with lower levels of education, and those in economically developed areas.

## Data Availability

The data that support the findings of this study are openly available at the following URL/DOI: http://charls.pku.edu.cn/.

## Abbreviations

NO_2_: Nitrogen dioxide
PM_10_: Particulate matter
EPI: Environmental performance index
AQI: Air quality index
API: Air pollution intensity
CHARLS: China health and retirement longitudinal study
ADL: Activity of daily living
CO: Carbon monoxide
OLS: The ordinary least squares regression
IADL: Instrumental activities of daily living
BADL: Basic activities of daily living
IRB: Institutional review board
OR: Odds ratio
CSI-D: Community screening instrument for dementia
CI: Confidence Intervals
LR: Likelihood ratio
ID: Identification documents
GDP: Gross domestic product
